# Transoral unilateral lag screw osteosynthesis for coronal split fracture of the lateral mass of the atlas – case report, operative technique and review of the literature

**DOI:** 10.1016/j.bas.2023.101761

**Published:** 2023-06-13

**Authors:** Christian Tinner, Fabian Cedric Aregger, Moritz Caspar Deml

**Affiliations:** Department of Orthopaedic Surgery and Traumatology, Inselspital, Bern University Hospital, University of Bern, Switzerland

**Keywords:** Atlas fracture, Minimally invasive, Transoral approach, Upper cervical spine

## Abstract

**Introduction:**

Atlas ring fractures, which account for 1.3% of all spinal fractures, are predominantly managed conservatively. However, in certain cases, surgical treatment may be necessary depending on the type of fracture, degree of comminution, fracture location, and associated ligamentous injuries. Surgical stabilization frequently results in a posterior C1-2 or C0-2 fusion, which restricts movement, particularly craniocervical rotation. Coronal split fractures of the lateral mass need to be reduced and fixed due to dislocation, instability and secondary osteoarthritis. The preferred treatment approach involves internal fixation of the reduced fracture fragments, while avoiding restriction of the upper cervical spine's range of motion (ROM).

**Research question:**

Is unilateral anterior transoral lag screw for treatment of unstable coronal split fracture of lateral mass of the atlas feasible and a safe treatment option?

**Case Report: Material and Methods:**

We report on a 55-year-old female suffering from polytrauma with multiple spinal and extremity injuries.

**Results:**

A coronal split fracture of the lateral mass of the atlas was treated minimally invasive with a transoral lag screw technique to reduce and fix the fracture that has a tendency for fracture gap widening. Stable fixation and fracture union and thus restoration of function was achieved.

**Discussion and conclusion:**

Transoral lag screw osteosynthesis for coronal split fracture of the lateral mass of the atlas is a potential treatment option in selected cases to preserve mobility in the upper cervical spine after spinal trauma.

## Highlights

• Is minimally invasive with less blood loss.
• Is a fast procedure.
• Avoids C0-2 arthritis and preserves rotational mobility in the upper cervical spine.

## 1 Introduction

Atlas fractures typically occur as a result of low-energy falls in elderly individuals, or high-energy trauma with axial loading in younger patients (Watanabe et al., 2010). They account for one-fourth of all craniocervical injuries, around 7% of cervical fractures, and 1.3% of all spinal fractures (Dickman and Sonntag, 1997; An, 1998). It was initially described and classified by Jefferson (G, 1919), later Segal et al. (1987), Levine and Edwards (1991), Landell (Landells and Van Peteghem, 1988), and Gehweiler (Gehweiler et al., 1976) depending on location and integrity of the transverse ligament. Recently the new AO classification for the upper cervical spine re-classified these injuries depending on stability and therapeutic consequences (Vaccaro et al., 2022).

The anatomy of the Atlas is unique. Thin anterior and posterior arches connect the two lateral masses. It lacks a spinal process and vertebral body. It developes from three ossification centers. An incomplete formation of the posterior arch is a relatively common anatomic variant (Junewick et al., 2011). In cervical trauma, the thin arches represent weak points and usually fracture at least twice. Burst fractures, posterior arch fractures, and comminuted lateral mass fractures represent 20–30% of all atlas injuries, respectively (Bransford et al., 2011). Prolonged union time with persistent neck pain is expected in anterior fractures of the atlas with an insufficient or ruptured transverse ligament (Easter et al., 2011; Guiot and Fessler, 1999; Wang et al., 2012). The risk of neurologic injury is low due to an ample space for the spinal cord at this level (Levine and Edwards, 1991; Han et al., 1976). The fracture location and integrity of the transverse ligament often determine the treatment strategy (Dickman and Sonntag, 1997; Landells and Van Peteghem, 1988; Vaccaro et al., 2022).

Computed tomography is the radiologic modality of choice to delineate fracture patterns and identify associated injuries in the cervical spine. It should be performed in every case of suspected cervical spine injury (Lin et al., 2003). For diagnosing additional ligamentous injuries, magnetic resonance imaging (MRI) is more sensitive (Spence et al., 1970; Jo et al., 2011; Haus and Harris, 2008).

Surgical stabilization usually consists of posterior C1/2 or occipitocervical (C0-2 or even C0–C3) fusion leading to a restricted range of motion (ROM) (Payer et al., 2009) and causing subaxial degenerative changes (Ruf et al., 2004). Posterior motion-preserving ring osteosynthesis techniques fixing the laterally displaced fragment in a reduced position as internal immobilization have been described (Jo et al., 2011; Ruf et al., 2004; Abeloos et al., 2011; Malcolm et al., 2017), but fail to address anterior unstable fractures directly. Anterior transoral motion-preserving ring osteosynthesis has been described with screw-plate or screw-rod constructs (Ruf et al., 2004; Hu et al., 2014; Ma et al., 2013; Zou et al., 2020; Keskil et al., 2017). Keskil et al. described a single case in which unilateral posterior lag screw osteosynthesis was used to treat an isolated, non-displaced anterior ¼ atlas fracture. The authors indicated osteosynthesis in potentially unstable fracture with delayed union and persisting pain to prevent arthrosis and pain caused by immobilization (Keskil et al., 2016). For the same reason other cases have been reported with posterior lag screw fixation of coronal split fractures (Felbaum et al., 2018; Minardi et al., 2022; Tabbosha et al., 2013).

This paper aims to describe the transoral lag screw technique for a displaced and potentially unstable coronal split fracture of the right lateral mass of the atlas with closed reduction and osteosynthesis of the right anterior ring of C1.

## 2 Material and methods

**Fig. 1:**
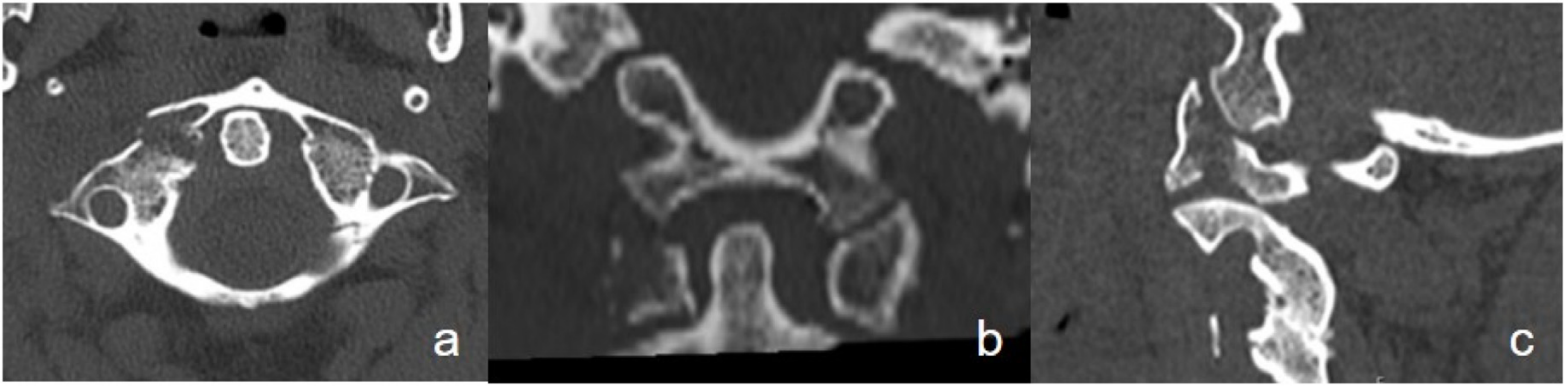
Preop CT scan of C1. a) axial, b) coronar, c) saggital plane.

A 55-year-old female experienced a 30m fall while rock climbing. She was diagnosed with polytrauma (ISS 17) abroad and was repatriated to our department. Her neurological examination was regular except for a Glasgow Coma Scale (GCS) of 14 points due to traumatic brain injury. She suffered from diffuse posterior cervical and thoracic tenderness. A whole-body CT-scan revealed a C1 fracture with a dislocated lateral mass fracture by 5 mm on the right side and a non-displaced posterior arch fracture on the left side. CT Angiography revealed no injury of the vertebral artery. The fracture was classified as C1/2: A-Type Fracture (N 0, M 1–3 according to AO Spine) (Vaccaro et al., 2022) and atypical Gehweiler Type IV with additional non-displaced posterior arch fracture (Gehweiler et al., 1976) (Fig. 1). Additionally, a minor traumatic brain injury, several rib fractures, burst fractures of the thoracic spine (Th 4: A3 and Th5: A4 according to AO spine), dislocated wrist fracture, and perilunate dislocation on the left side were diagnosed. Initially, the dislocated wrist and carpus were fixed, and the spine was immobilized with a stiff collar and bed rest.

We recommended surgical stabilization of the cervical and thoracic spine in a neurological stable situation. To achieve stability of the C1 ring, posterior arch fixation alone would be difficult to address the anterior fracture component with the risk of inadequate repositioning and compression of the fracture fragments. A more invasive option for achieving stability bot not prevent late arthritis in C0/1 would be posterior C1/2 Goel-Harms fixation (Harms and Melcher, 2001). Therefore, we decided to achieve proper compression of the fragments, stabilization of the C1 ring, and allow motion preservation in the upper cervical spine by an anterior right-sided transoral lag screw fixation of the C1 ring. The posterior part of the fracture was stable and not displaced. It was mostly stabilized by non-displaced periost.

### 2.1 Surgical technique

**Fig. 2:**
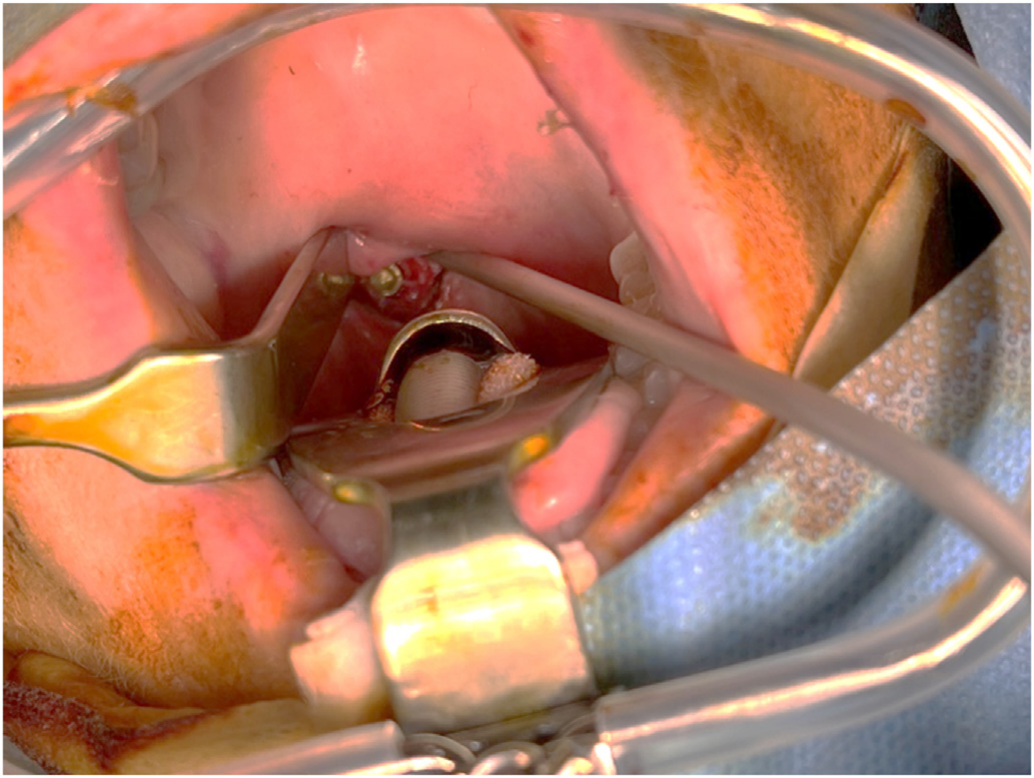
Exposure with self-retractor with screw in situ after stabilization.

After informed consent, the patient was under general anesthesia in a supine position with slight reclination of the skull secured by a Mayfield clamp (Integra Co, Plainsboro, New Jersey, USA). A single shot of parenteral antibiotics was given prophylactically. The surgeon stood on the patient's right side and disinfection with betaseptic around the mouth was performed. Next, a self-retaining oral retractor, usually used for tonsillectomy, was placed to visualize the soft palate, followed by repeated disinfection enorally with betadine (Fig. 2). After a standardized team-time-out, we localized the entry point with a 2.0mm k-wire under fluoroscopy in two planes.

**Fig. 3:**
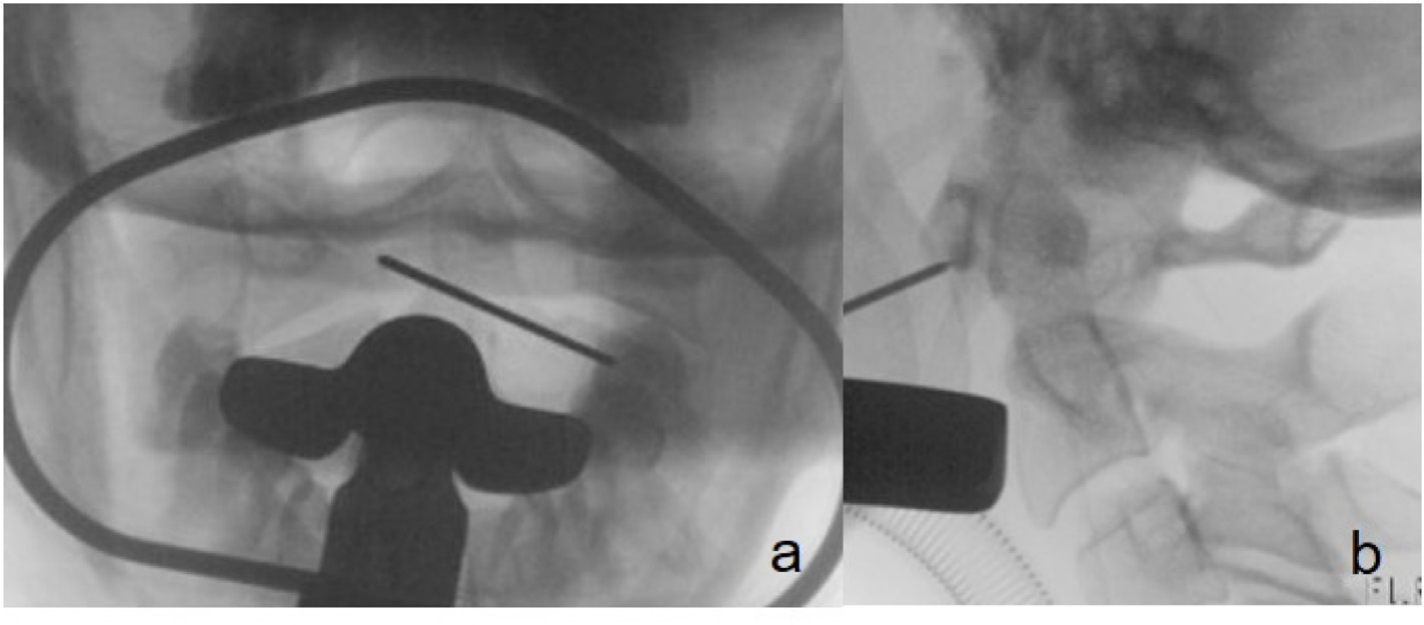
Ap/lateral fluoroscopy to mark the entry point with a k-wire.

**Fig. 4:**
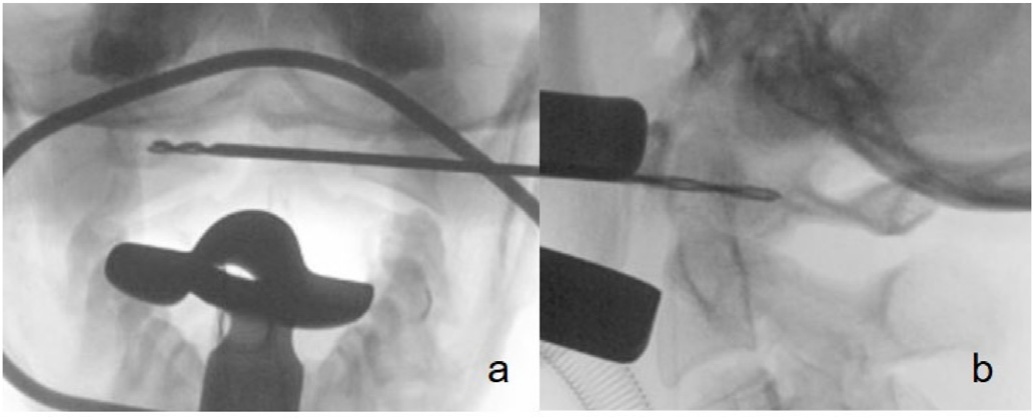
Ap/lateral fluoroscopy to verify the correct drill position for the screw.

**Fig. 5:**
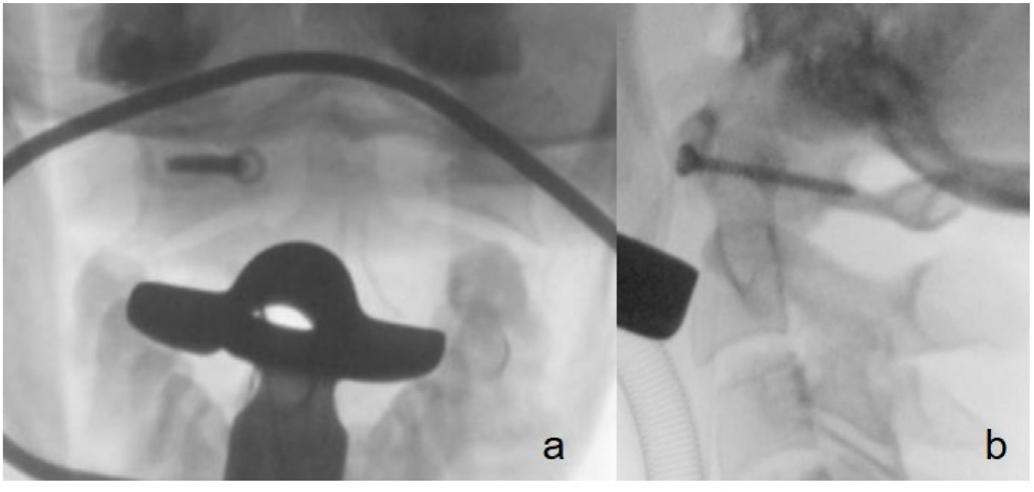
Ap/lateral fluoroscopy with the placed screw.

**Fig. 6:**
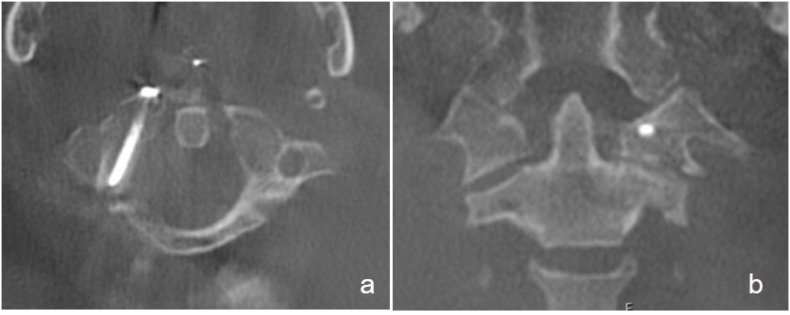
Intraoperative 3D scan with C-arm to confirm correct reposition and screw-position axial (a) and coronal (b).

Once the correct position was verified fluoroscopically, we cut the mucous membrane over about 8mm. We dissected the soft tissue bluntly to the anterior part of the C1-ring, using a dissector and bipolar coagulation. The entry point was localized again with a k-wire and verified under fluoroscopy in two planes (Fig. 3). Next, the cortical bone was perforated using a 2.0mm drill. The fracture gap was palpable while drilling into the posterior fragment. The fluoroscopy in two planes showed the correct position of the drill (Fig. 4). After over-drilling the proximal cortex with 2.7mm for gliding, a 26 ​× ​2.7mm cortical screw was inserted and tightened with excellent purchase. The fracture gap was closed (Fig. 5). Irrigation of the situs with betadine and closure of muscular and mucosal tissue with vicryl rapid 3.0 single stitches were performed. After removing the retractor, we verified the fracture gap's correct screw placement and closure with an intraoperative 3D scan (Ziehm Vision RFD 3D, Ziehm Imaging GmbH, Germany) (Fig. 6). The procedure including exposure and fracture stabilization took 35 minutes with a minimal blood loss of less than 20 ml. The procedure was followed by open spondylodesis Th3/4 – Th7/8 to stabilize the burst fractures Th5/6.

### 2.2 Postoperative course

The patient developed pharyngeal swelling leading to delayed extubation and resulting in surveillance in the intensive care unit. Two days later, the craniomaxillofacial surgeons performed an osteosynthesis of the viscerocranium. Due to facial injuries and swollen airways, a tracheostomy was established. Subsequently, the patient could be transferred to a regional hospital, followed by neuro-rehab. Tracheostomy was removed.

**Fig. 7:**
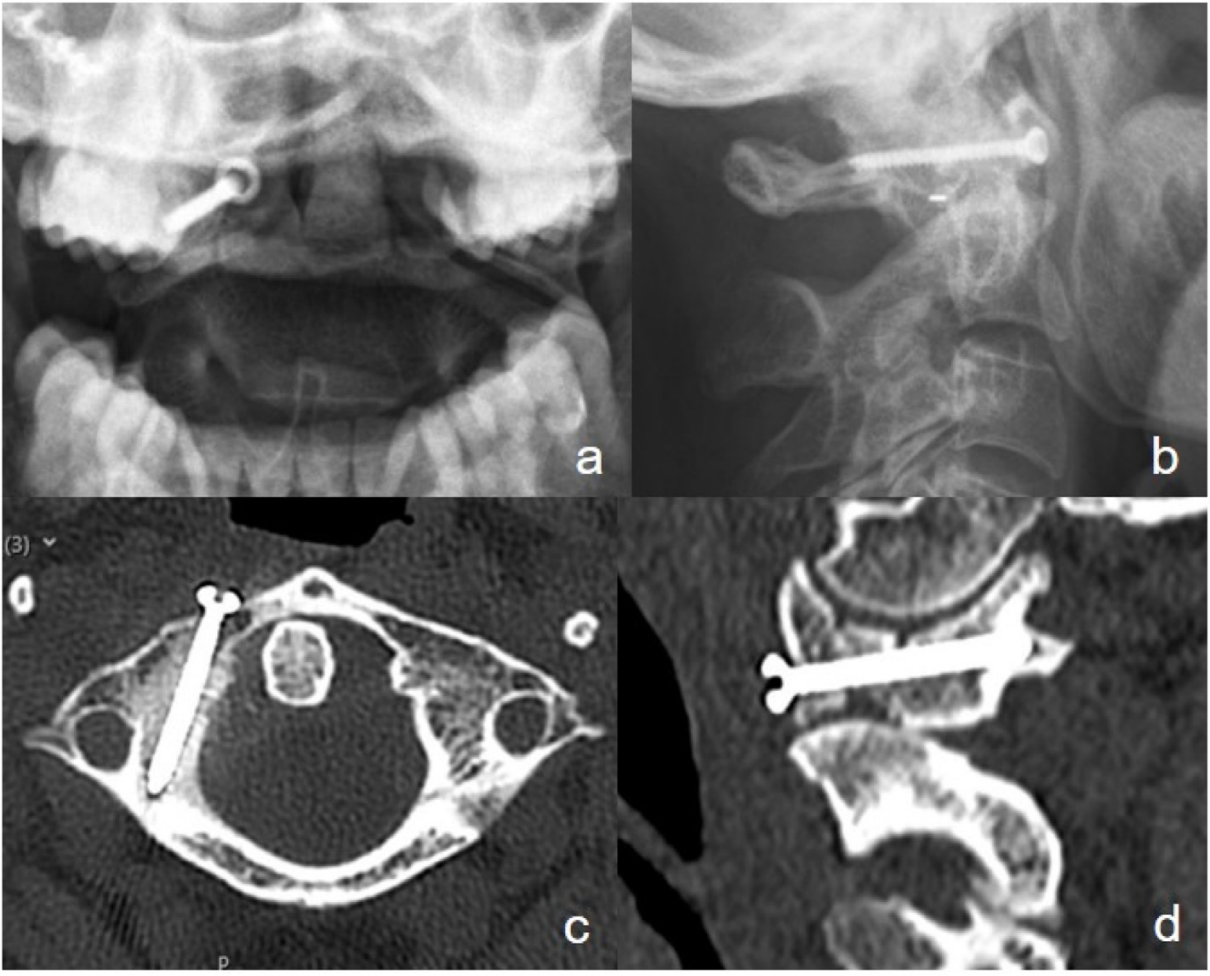
Follow-up x-ray (a/b) and CT-scan (c/d) after six weeks with correct screw position and no loss of reduction.

**Fig. 8:**
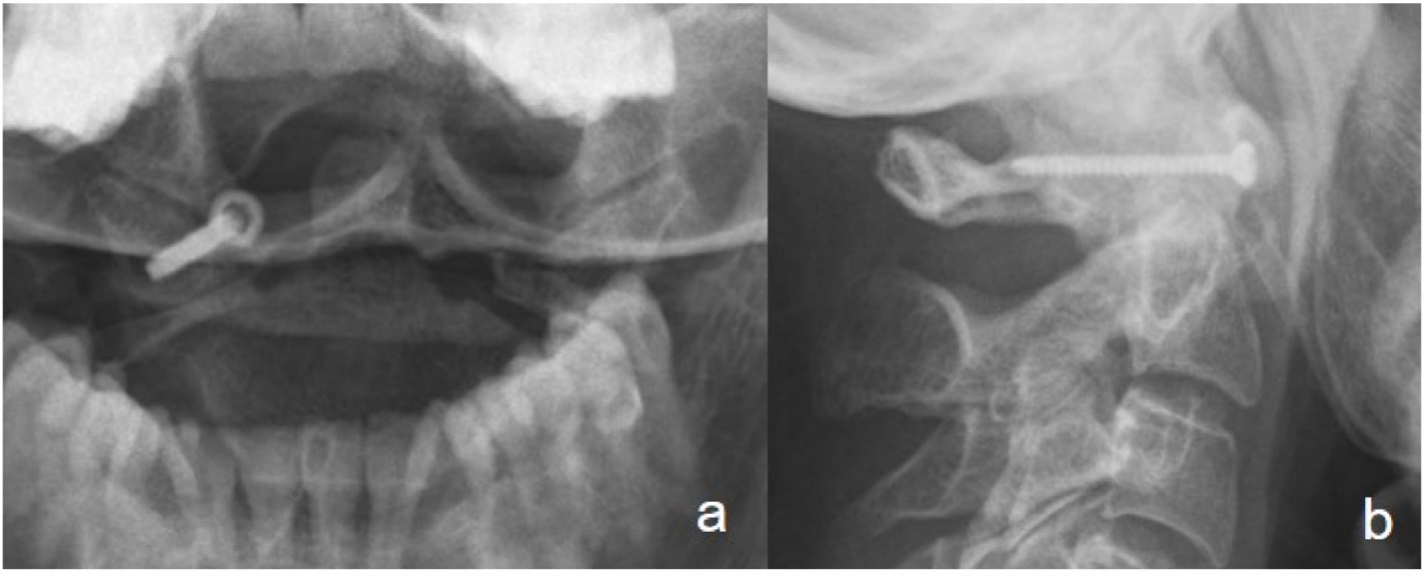
Follow-up x-ray (a/b) after 6 months with the unchanged position.

The first follow-up after six weeks showed a pain-free patient with a radiologically satisfactory result. In addition, the CT scan showed anatomical reduction and stable fracture fixation (Fig. 7). Therefore, we replaced the rigid collar with a soft one for four more weeks. At the next follow-up visits after 12 weeks and 6 months, the patient remained pain-free with nearly physiological movement conducting ambulatory physical therapy (Fig. 8).

## 3 Discussion

Transoral approaches have been known for years, with declining complication rates to technical improvement (Apuzzo et al., 1978; Crockard, 1985; Dickman et al., 1992; Menezes and VanGilder, 1988; Tuite et al., 1996). Although neck pain persists in 20–80% of the patients after external immobilization (Segal et al., 1987), conservative treatment is still state of the art for most of C1 fractures with satisfactory radiological and clinical results. In unstable or dislocated fractures, surgery should be performed. Posterior C1/2 or C0/2 fusion was first described in 1910 (Mixter and Osgood, 1910), and technological improvement over the past decades showed reliable and satisfactory results with screw-rod systems, according to Goel/Harms (Payer et al., 2009; Harms and Melcher, 2001). The major disadvantage of this technique is the resulting restriction in ROM in the crucial articulation of C1/2 for rotation and C0/1 for flexion/extension (Payer et al., 2009). C1/2 accounts for up to 75% of rotational motion in the upper cervical spine (Salem et al., 2013). Additionally, an increased incidence of degeneration of the subaxial cervical spine has been reported after C1/2 fixation (Ruf et al., 2004).

An isolated C1 ring osteosynthesis allows stabilization of the fracture and preservation of joint motion and potentially diminishes the degeneration progress associated with fusion. The transoral approach with C1-ring osteosynthesis has been reported with screw-wire or plating systems in anterior ring fractures (Ruf et al., 2004; Zou et al., 2020; Keskil et al., 2017). Others also report on posterior unilateral lag screw technique for slightly displaced anterior C1-ring or coronal split fractures of the lateral mass to prevent post-traumatic degenerative changes and further dislocation (Keskil et al., 2016; Felbaum et al., 2018; Minardi et al., 2022; Tabbosha et al., 2013; Lakshmanan et al., 2005). By open posterior approach, irritation of the C2 nerve root can occur (Harms and Melcher, 2001), and the venous plexus is at risk in addition to often reported postoperative pain and muscular irritations (Blagg et al., 2009; Gunnarsson et al., 2007; Rhee et al., 2008). Navigation could simplify screw placement and facilitate a percutaneous approach, with comparable risk to crucial structures. However, in our view, navigation in highly unstable fracture situations can be challenging and potentially misleading due to a high potential for inaccuracy. Kandziora et al. described safe zones for screw placement in the atlas and axis, which could facilitate free-hand placement with increased safety (Kandziora et al., 2001).

Our report presents a technique for reducing and stabilizing an unstable anterior arch fracture of the atlas. The method involves using an anterior lag screw to achieve union, prevent further dislocation and late arthritis and maintain C0/1 and C1/2 motion. Similar techniques have been described from a posterior approach (Keskil et al., 2016; Felbaum et al., 2018; Minardi et al., 2022; Tabbosha et al., 2013, Farrokhi et al., 2019). To the best of our knowledge, this is the first case report of using a single anterior lag screw to reduce and stabilize an coronal split fracture of the lateral mass of the atlas by an anterior transoral approach. Main advantages of the anterior transoral approach include reduced postoperative pain and faster exposure with shorter surgical time, which was in our polytrauma case important. However, a limitation is a risk of postoperative swelling of the pharynx and delayed extubation, which has been reported in 4.2% of cases (Amelot et al., 2018). Additionally, anterior transoral approaches may lead to severe implant-associated infections with defects in the posterior pharyngeal wall, which can require extensive surgical reconstruction. Nevertheless, the minimally invasive approach with a stab incision and low screw surface may reduce the risk of postoperative implant-related complications and infections. Intraoperative 3D imaging showed high potential to secure the fracture reposition and correct screw position.

## 4 Conclusion

Transoral anterior lag screw fixation for coronal split fractures of the lateral mass of the atlas is a potentially effective surgical option. In our case, the anatomic reduction was achieved with bony fusion and preserved rotational and flexion/extension motion in the critical C0/C1 and C1/C2 segments.

## Declaration of competing interest

We declare no conflicts of interest.
