# Supplementary material for: Transoral unilateral lag screw osteosynthesis for coronal split fracture of the lateral mass of the atlas – case report, operative technique and review of the literature
Source: Brain Spine. 2023 Jun 13;3:101761. doi: 10.1016/j.bas.2023.101761 (PMC10668072; doi:10.1016/j.bas.2023.101761)
Supplement: Multimedia component 1 [file mmc1.docx]

**Review of the Literature**

| **Year; Journal** | **Title** | **Authors** | **N° of cases** | **Approach, Fixation** |
| --- | --- | --- | --- | --- |
| 2004; Spine | Transoral Reduction and Osteosynthesis C1 as a Function-Preserving Option in the Treatment of Unstable Jefferson Fractures | Ruf M, Harms J et al. | 6 | Anterior transoral, plate-screw or screw-rod |
| 2013; Eur Spine J | Unstable atlas fracture treatment by anterior plate C1-ring osteosynthesis using a transoral approach. | Ma, W., et al. | 20 | Anterior transoral, plate-screw |
| 2013; J Neurosurg Spine | Placement of unilateral lag screw through the lateral mass of C-1: description of a novel technique | Tabbosha M., et al. | 1 | Posterior, lag screw |
| 2014; Indian J Orthop | Unstable Jefferson fractures: Results of transoral osteosynthesis | Hu Y, Dong W et al. | 12 | Anterior transoral, plate-screw |
| 2016; J of Craniovertebr Junction Spine | Unilateral lag-screw technique for an isolated anterior 1/4 atlas fracture | Keskil S, Yüksel U et al. | 1 | Posterior, lag screw |
| 2017; J of Craniovertebr Junction Spine | Transoral screw and wire fixation for unstable anterior ½ atlas fracture | Keskil S, Yüksel U et al. | 1 | Anterior transoral, screw-wire |
| 2017; World Neurosurgery | Unilateral C1 Sagittal Split Fractures: An Unusual Entity Revisited | Felbaum DR et al. | 2 | Posterior, lag screw |
| 2019; Br J of Neurosurg | Unilateral lag screw fixation of isolated non-union atlas lateral mass fracture: a new technical note | Farrokhi, M.R et al. | 1 | Posterior, lag screw |
| 2020; BMC Musculoskelet Disord | Motion-preserving treatment of unstable atlas fracture: transoral anterior C1-ring  osteosynthesis using a laminoplasty plate | Zou X, Ma X et al. | 13 | Anterior transoral, plate-screw |
| 2021; BMC Musculoskelet Disord | Anterior reduction and C1-ring osteosynthesis with Jefferson-fracture reduction plate (JeRP) via transoral  approach for unstable atlas fractures | Tu Q, Xia H et al. | 22 | Anterior transoral, plate-screw |
| 2021; Eur Spine J | Direct repair of displaced anterior arch fracture of the atlas under microendoscopy: experience with seven patients | Wang J, Liu J et al. | 7 | Anterior endoscopic with autograft |
| 2022; Brain and Spine | Lag screws for reduction of bilateral lateral mass fractures due to spinal trauma | Minardi M, et al. | 1 | Posterior, bilateral lag screws |

**Table 1:** Overview of the literature for isolated osteosynthesis of the Atlas with anterior approach or lag screw technique.

| **Approach** | **N° of articles** | **N° of cases** |
| --- | --- | --- |
| Anterior transoral, plate-screw / screw-rod | 6 | 74 |
| Posterior, lag screw | 5 | 6 |
| Others | 1 | 7 |

**Table 2:** Summary of the publications and cases
